# Relationship between non-communicable diseases and background characteristics among homeless people in Nagoya City, Japan

**DOI:** 10.1371/journal.pone.0219049

**Published:** 2019-07-05

**Authors:** Akihiro Nishio, Ryo Horita, Tadahiro Sado, Takahiro Watanabe, Ryosuke Uehara, Seiko Mizutani, Mayumi Yamamoto

**Affiliations:** 1 Health Administration Center, Gifu University, Gifu, Japan; 2 Department of Psychopathology, Division of Neuroscience, Graduate School of Medicine, Gifu University, Gifu, Japan; 3 Faculty of Health Promotional Sciences, Tokoha University, Hamamatsu, Japan; 4 Midori Hospital, Gifu, Japan; 5 Yoshida Hospital, Nara, Japan; 6 Faculty of Nursing, Nihon Fukushi University, Miyahama-okuda, Aichi, Japan; 7 United Graduate School of Drug Discovery and Medical Information Sciences, Gifu University, Gifu, Japan; University of Auckland, NEW ZEALAND

## Abstract

**Background:**

There are few reports that objectively show actual health conditions among the homeless or diagnoses of non-communicable diseases based on blood tests. This report discloses the actual data from blood tests and non-communicable diseases among the participants. Furthermore, associations between the test values for lifestyle-related disease and mental disorder/intellectual disability, as well as personal backgrounds of homeless people, were analyzed from the data gathered in the survey.

**Methods:**

This study was performed in a rented meeting room close to Nagoya Station on November 2, 2014. Blood samples, physical measurements, blood pressure measurements intellectual faculties were measured. Physical/mental diseases were diagnosed by doctors. Fisher’s exact test was performed to compare between subgroups (by participants’ socio-demographic data or the presence of mental illness/cognitive disability) according to non-communicable disease test values, and to calculate the odds ratio.

**Results:**

Abnormalities among participants in test values for non-communicable disease were as follows: hypoalbuminemia in one participant (0.9%), abnormalities in liver function in 22 participants (19.3%), decreased renal function in two participants (1.8%), dyslipidemia in 67 participants (58.8%), "a person whose impaired glucose tolerance cannot be ruled out" in 20 participants (17.5%), obesity in 33 participants (28.9%), thinness in five participants (4.3%), and hypertension in 60 participants (52.6%). Compared to the National health and nutrition survey 2015, non-communicable diseases of the homeless people were similar or slightly better than in the general population in Japan. Participants aged 20–39 years had a significant positive tendency of having liver function abnormality compared to ≥60 years old participants. There was no significant tendency with socio-demographic characteristics in dyslipidemia and “impaired glucose tolerance”.

**Conclusion:**

It was found that the percentage of homeless people in Nagoya who showed abnormalities of lifestyle-related disease was similar or better than that of general population in Japan.

## Introduction

It has often been reported that the morbidity and prevalence of tuberculosis are high in homeless populations. In addition, two surveys in Osaka District in Japan revealed that homeless people often suffer from malnutrition [1, 2]. However, there are few reports that objectively show the actual health conditions among the homeless or that diagnosed non-communicable diseases based on blood test results. Bernstein conducted a systematic review and meta-analysis regarding diabetes and hypertension prevalence in homeless adults in the United States [3]. The review consisted of 53 articles that reported the prevalence of diabetes or hypertension. However, almost all surveys identified patients with diabetes based on self-report and only three articles diagnosed diabetes using laboratory data. These three articles targeted homeless people who visited medical clinics or the hospitals. Therefore, all three articles did not report the prevalence of diabetes among the general homeless population. As far as we know, only two reports from Western countries and one from Japan showed the actual prevalence of non-communicable diseases, including diabetes, hypertension, liver function abnormalities, dyslipidemia, and decreased renal function, using blood test results. One was reported by Arnaud et al. [4], and targeted 488 persons (80% were men) who were lodged at nine Paris shelters in France, and measured capillary glycemia followed by venous glycemia. Diabetes was diagnosed if capillary glycemia was ≥120 mg/dl or ≥150 mg/dl after fasting for <8 h, with an estimated diabetes prevalence of 6.2%. The other article was by Scott et al. [5]. This survey targeted 156 homeless men (mean age 47.2 ± 14.6 years) and 96 homeless women (mean age 39.1 ± 13.5 years), who used homeless services in Ireland. Of the 252 participants, 8%, 10%, and 21% were diagnosed with type 2 diabetes, pre-diabetes, and the metabolic syndrome, respectively. It was reported that 44%, 27%, 87%, and 18% had hypercholesterolemia, hypertriglyceridemia, raised low-density lipoprotein (LDL), and reduced high-density lipoprotein (HDL) cholesterol, respectively. The researchers concluded that the prevalence of diabetes, pre-diabetes, and metabolic syndrome in the homeless population was similar to the national estimates. Kuroda [6] conducted a survey in Japan that targeted 917 homeless people aged over 55 years, who were registered in the career department that provided jobs for poor people. This survey reported that 13.0%, 10.7%, 29.3%, 3.3%, 33.7%, and 1.7% of participants had abnormal values for aspartate transaminase (AST), alanine aminotransferase (ALT), total cholesterol, HDL, triglyceride (TG), and albumin, respectively. The cut-off systolic and diastolic blood pressure measurements in participants with hypertension were >140 mmHg and >90 mmHg, respectively with 58.1% and 45.5% of participants reported as having abnormal values. Few reports exist on this topic and the previous surveys used varied target population or standard and therefore it was difficult to generalize the findings. Therefore, we should focus on accumulating data to better understand the homeless population.

To address this topic, we conducted a survey that included the following: non-communicable diseases including psychiatric disorders, measurement of intellectual faculties, and interviews regarding people’s personal histories and the causes of homelessness, in 114 homeless people living in Nagoya City. We already reported higher prevalence of mental illness and cognitive disability among the Japanese homeless population [7, 8] and causes of homelessness prevalence [9, 10]. In this previous study, we reported the actual blood tests and non-communicable diseases data among the participants. Furthermore, associations between the test values for non-communicable disease and mental disorder/intellectual disability, as well as personal backgrounds of homeless people, were determined from the analysis of data gathered in the complex survey.

Nagoya is the fourth biggest urban city with a population of approximately 2.3 million people in Japan. Free physical examination and psychiatric consultation have been provided to homeless people since 1985 by Sasashima Support Center, which is a non-governmental organization (NGO) in Nagoya. From a 30-year experience, it has been realized that only a few homeless people could escape their situation and those who do, sometimes return to their street life, despite the availability of social welfare for homeless people in Japan.

According to Nagoya City’s report [11], there were 211 street people in January 2016. This report also showed the number of street people in January 2013 (305), 2014 (264), and 2015 (273). A downward trend can be seen in Nagoya. However, Nagoya city’s report counted only street people without including homeless people who lived in public or private facilities (e.g., shelters) or in transitional housing. Therefore, the total number of homeless people in Nagoya was not known.

The aim of this study was to determine the prevalence of non-communicable diseases or abnormalities and its relationship with socio-demographic characteristics among Japanese homeless people.

## Methods

### Definition of homelessness

The Japanese government defined a homeless person as an individual who takes up residence in a city park, riverbank, roadside, station building, or other places to live without a reason [12]. However, for this study, we adopted the definition of the US Department of Health and Human Services, which referred to someone who is “homeless” as “an individual who lacks housing without regard to whether the individual is a member of a family, including an individual whose primary residence during the night is a supervised public or private facility (e.g., shelters) that provides temporary living accommodations, and an individual who is a resident in transitional housing” [13].

### Research design

This study was a cross-sectional survey. It was performed in a rented meeting room close to Nagoya Station on November 2, 2014. In this study, homeless people were recruited with the cooperation of the Sasashima Support Center, a social welfare NGO that specializes in homeless support, covering the entire Nagoya area. The survey was advertised for 2 months at the meal service place or during the NGO’s daily activities, such as rounds at night or during medical or daily life consultations. Participants were provided a 3000-yen prepaid card after the survey.

Blood sampling for liver function tests (AST, ALT, and γ-glutamyl transpeptidase [γ-GTP]); serum lipid (including HDL-cholesterol, LDL-cholesterol, and TG), creatinine (Cre), uric acid, albumin, and hemoglobin A1c (HbA1c) were conducted. Blood pressure, height, and body weight were also measured. Body mass index (BMI) was calculated as body weight (kg)/[height(m)]^2^. BMI was categorized into obesity (>25) and thinness (<18.5) according to Japanese criteria for obesity [14]. Hypertension was diagnosed if the systolic blood pressure was >140 mmHg or the diastolic blood pressure was >90 mmHg, according to Japanese hypertension guidelines [15]. Hypoalbuminemia was defined as albumin <3.7 g/dl. Liver function abnormalities occurred with AST >40 U/l; ALT >45; γ-GTP and >49 U/l for male and female, respectively. HDL <40 mg/dl, LDL >139 mg/dl, or TG >149 mg/dl were considered as dyslipidemia according to Japanese diagnostic criteria for dyslipidemia [16]. Hyperuricemia was defined as uric acid (UA) >7.0 mg/dl. HbA1c >5.9%, was regarded as “impaired glucose tolerance” based on Japanese diagnostic criteria for diabetes [17]. Estimated glomerular filtration rate (eGFR) was calculated as follows: 194 × (Serum Cre)^-1.094^ × age^-0.287^ for men; and 194 × (Serum Cre)^-1.094^ × age^-0.287^ × 0.739 for women; if eGFR <60, decreased renal function was considered.

### Participants’ socio-demographic and background characteristics

Participants’ age, residence status, duration, and history of homelessness, alcohol consumption, drug use, tobacco smoking, gambling, social support history, pension status, and educational levels were obtained by medical professionals via interviews.

### Diagnosis of mental illness

The psychiatrists conducted semi-structured interviews on the participants using the Mini-International Neuropsychiatric Interview according to the diagnostic criteria of the Diagnostic and Statistical Manual of Mental Disorders, Fourth Edition, Text Revision (DSM-IV-TR) [18].

### Diagnosis of cognitive disabilities

Clinical psychologists assessed each participant’s current cognitive capacity using the simplified version of the third edition of the Wechsler Adult Intelligence Scale (WAIS-III). This study used the method by Dairoku et al. [19], which consists of 4 WAIS-III subtests (Picture Completion, Digit Symbol-Coding, Digit Span, and Information) out of 13. Total intelligence quotient (IQ) was calculated by doubling 4 total scores and adding 20 points. We considered individuals with IQ below 70 as having cognitive disabilities.

### Statistical analyses

Fisher’s exact test was performed to compare between subgroups (by participants’ socio-demographic data or the presence of mental illness/cognitive disability) according to non-communicable disease test values, and to calculate the odds ratio. Statistical analyses were performed using JMP1 ver. 10.0.2 (SAS Institute, Tokyo, Japan). Participants were classified into two groups based on the presence or absence of abnormalities according to non-communicable diseases test results. Then, they were classified into two or three subgroups based on participants’ background characteristics including gender, age, place of residence, education, duration of homelessness, pension, alcohol consumption, smoking, gambling, or the presence of mental disorder/cognitive disability. The control group was defined by the following parameters; male versus female; those aged 20–29 years versus 40–59 years versus >60 years old; those living in the street versus living in a shelter or any temporary place; homelessness for less than 1 year versus 1–4 years and >4 years duration; those who had a pension versus having no pension; those who did not smoke versus those who smoked 1–20 cigarettes, and those who smoked >20 cigarettes per day; those who did not gamble versus those who constantly gambled, and those who gambled sometimes; those without mental illness versus those with mental illness; and those without intellectual disabilities versus those with intellectual disabilities.

### Ethical statement

The Ethical Review Committee of Gifu University’s Graduate School of Medicine approved this research protocol on August 6, 2014 (approval No. 26–133). All participants received detailed face-to-face explanations regarding the protocol before providing their consent. The consent form was written in an easy-to-read manner. All participants gave written informed consent. On humanitarian grounds, participants who required medical care or welfare services based on the interviews and their medical records were referred to the appropriate medical institutions at the time of the survey.

## Results

### Participants’ socio-demographic and mental problem

We showed the background characteristics of participants in Table 1. Of 114 participants (106 men and 8 women) enrolled in this study, all were Japanese, and their ages ranged from 20 to 78 years (mean age, 54.0 ± 12.6 [SD] years). Seventy-two participants lived in the street, 35 lived in temporary residence, while 7 either had no response or reported living in other situations such as staying at an internet café. The duration of current homelessness ranged from <1 to 20 years (mean duration, 3.5 ± 4.4 years). Thirteen subjects received old age disability or other pensions. Seventeen participants took alcohol of >2 l of wine per week (calculated from the alcohol content). There was no drug user. Eighty-one participants were regular smokers. Thirty-eight subjects gambled regularly and fifty had previous experience of gambling. Of 48 (42.1%) participants that were diagnosed with a mental illness: 4.4%; 17.5%; 2.6%; 14.0%; 3.5% had schizophrenia or other psychotic disorder; mood disorder; anxiety disorder; substance-related disorder; and personality disorder, respectively. Cognitive disability affected 34.2% of the participants. The overlap between mental illness and cognitive disability was 15.8%. Detailed mental problems among homeless people were already published [7].

**Table 1:** Background characteristics of participants.

Gender	Male	Female	
	106 (93.0%)	8 (7.0%)	
Age (years)	20–39	40–59	60 or more
	18 (15.8%)	53 (46.5%)	43 (37.8%)
Residence	Street	Temporary housing	Other
	72 (63.1%)	35 (30.7%)	7 (6.1%)
Education^#^	Grade 9	Grade 12	Graduated University
	62 (55.3%)	44 (39.3%)	6 (5.3%)
Duration of homelessness	less than 1 year	1–4 years	more than 5 years
	66 (57.9%)	22 (19.3%)	26 (22.8%)
Pension	No	Yes	
	101 (88.6%)	13 (11.4%)	
Alcohol consumption (number of 200 ml wine bottles/week)	Do not drink	1–9	more than 10
	69 (60.5%)	27 (23.7%)	17 (14.9%)
Smoking (number of cigarettes/day)	Do not smoke	1–20	more than 20
	33 (28.9%)	73 (64.0%)	8 (7.0%)
Gambling	Gamble sometimes	Stopped	Never do
	38 (33.3%)	50 (43.9%)	26 (22.8%)
Mental illness	Yes	No	
	48 (42.1%)	66 (57.9%)	
Cognitive disability	Yes	No	
	23 (20.2%)	91 (79.8%)	

^#^Sum of all category of education is 112

### Non-communicable diseases

We showed non-communicable diseases and abnormalities in Table 2. Hypoalbuminemia, liver function abnormality, decreased renal function, hyperuricemia, dyslipidemia, “impaired glucose tolerance,” obesity, thinness, and hypertension occurred with 0.9%, 19.3%, 1.8%, 8.8%, 58.8%, 17.5%, 28.9%, 4.4%, and 52.6% of 114 participants, respectively. More detailed results were reported with high ALT (11.4%), high AST (14.0%), high γ-GTP (7.9%), low LDL (15.8%), high LDL (22.8%), and high TG (43.0%).

**Table 2:** Non-communicable diseases and abnormalities.

Disease or abnormality	
Hypoalbuminemia, n (%)	1(0.9%)
Liver function Abnormality, n (%)	22 (19.3%)
Decreased renal function, n (%)	2 (1.8%)
Hyperuricemia, n (%)	10 (8.8%)
Dyslipidemia, n (%)	67 (58.8%)
Impaired glucose tolerance, n (%)	20 (17.5%)
Obesity, n (%)	33 (28.9%)
Thinness, n (%)	5 (4.4%)
Hypertension, n (%)	60 (52.6%)

### Relationship between non-communicable disease and participants’ socio-demographic

We determined the relationship between participants socio-demographic and 5 non-communicable diseases (liver function abnormality, dyslipidemia, impaired glucose tolerance, obesity, and hypertension) if there are more than 10 participants. In S1 Table, the results of the relationships showed that participants aged 20–39 years had a significant positive tendency of having liver function abnormality compared to those aged ≥60 years. Further, sometimes gambled or never gambled participants had similar significant tendency of liver function abnormality. There was no significant tendency with socio-demographic characteristics in dyslipidemia and “impaired glucose tolerance.” Women had a significant tendency of having obesity compared to men. Furthermore, those aged 20–39 years had similar significant tendency compared to either those aged 40–59 or ≥60 years in having obesity. Men had a significant tendency of having hypertension compared to women. Participants aged ≥60 years had similar significant tendency compared to 20-39-year-olds. Furthermore, compared to those with <1 year of homeless experience, the prevalence of hypertension was significantly higher in participants with >5 years of homeless experience. Participants who drank more than 10 Japanese sake unit (it is nearly equal to 10 grams of pure alcohol) per week had similar significant tendency of having hypertension compared to non-drinkers.

### Relationship between non-communicable disease and mental illness/cognitive disability

The results showing the relationship between non-communicable disease and mental illness/cognitive disability are shown at the end of Table 3. There was no significant tendency with any of the combinations between non-communicable disease and mental illness/cognitive disability. In other words, we found no effect of illness/cognitive disability on non-communicable diseases.

**Table 3:** Comparison of the surveys.

	Our Survey	Kuroda et al.	Arnaud et al.	Scotto et al.
Conutry	Japan	Japan	France	Ireland
Year	2014	2003	2006	No data
Targeted people	General homeless	Homeless people who registered for job delivery service for elder homeless people	Homeless people lodged at Paris shelters	Homeless service users
Number of targeted people	114	917	488	252
Mean age (years)	54.0 ± 12.6	60.5 ± 3.5	No data	47.2 ± 14.6 (men) 39.1 ± 13.5 (women)
Gender (percentage of men)	93%	99%	96%	62%
Prevalence of obesity (BMI >25)	28.9%	11.2%	31.1%	53%
Prevalence of thinness (BMI <18.5)	4.4%	15.7%	7.5%	No data
Prevalence of diabetes	17.5% (Impaired glucose tolerance)	No data	6.2%	8%
Prevalence of hypertension	52.6%	58.1% (systric blood pressure >140mmHg) 45.5% (diastolic blood pressure >90mmHg)	35.2%	35%
Prevalencd of dislipidemia	58.8%	29.3% (Hypercholesterolemia, Total cholesterole > 220mg/dl) 33.7% (Hypertriglyceridemia, Triglycerides > 139mg/dl)	No data	44% (Hypercholesterolemia) 27% (Hypertriglyceridemia)
Liver function abnormality	19.3%	13% (AST > 41IU/l) 10.7% (ALT > 41IU/l)	No data	No data

## Discussion

This study presents the findings of a novel report showing the current non-communicable disease status or laboratory findings of the homeless in Japan. To the best of our knowledge, only two Western country reports [4, 5] and one from Japan [6] showed the actual prevalence of non-communicable diseases in the homeless. To compare our survey with those three articles, we summarized and compared the results of all four surveys as shown in Table 3. These four surveys differed in the target population, by age, gender, method of diagnosis, and in the definition of abnormality of life-related disease. Therefore, we may not be able compare our survey with other surveys. Regarding obesity, the prevalence of our survey was higher than that in the Kuroda survey but lower than that of Arnaud et al. and Scott et al.

It is often said that malnutrition is prevalent in the homeless Japanese [7, 8]. However, according to our study, there was only one participant who had hypoalbuminemia (3.5 g/dl), which although not very low, is one of the criteria for malnutrition. Similarly, those diagnosed with thinness were only 5 (4.4%), with the lowest BMI of participants being 17 kg/m^2^, which was also not very low.

Contrary to common sense of malnutrition among the homeless in Japan, in Western countries, obesity was more common [20–23]. Our survey also showed a high prevalence of obesity, “impaired glucose tolerance,” and dyslipidemia similar to the surveys of Arnaud et al. and Scott et al. However, both studies reported that there was no significant difference in the prevalence of diabetes between homeless people and the general population of France and Ireland, respectively.

To assess whether the prevalence of non-communicable diseases among Japanese homeless was higher compared to the Japanese general population, we compared our data with that provided by the Japan National health and nutrition survey 2015 [24]. According to that survey, the percentage of people with “impaired glucose tolerance” (> 5.9% of HbA1c) was 25.9% in adult males and 18.5% in adult females in the general population. The HbA1c result (17.5%) for homeless people (mostly men), was lower than that in the general population. Similarly, the national health and nutrition survey reported prevalence of hypertension in the general population (58.4% in adult males and 45.1% in adult females) was very similar to ours. Since the national health and nutrition survey defined dyslipidemia only for low HDL, which was self-reported, we therefore compared our data with Kuwahara et al. [25] workers’ data, with similar criteria as ours. According to that report, the prevalence of dyslipidemia among men and women aged 50–59 years were 51.2% and 42.7%, respectively. This percentage was almost similar to ours. The national health and nutrition survey also reported the prevalence of obesity and thinness among the general population as 28.6% and 4.7% in men, and 21.5% and 12.3% in women, respectively. These percentages were very similar to ours. Although some differences in conditions occurred, prevalence of non-communicable diseases among the homeless people in Japan were similar or slightly better than those in the general Japan population. Lee et al. [26] reported that hypertension, high cholesterol, and diabetes among Canadian homeless people were not more prevalent than in the general population. Our survey showed similar result.

Tahara et al. [27] obtained food records from 299 homeless men (mean age 57.6 ±8.0 years) and reported the total energy from the daily dietary intake. According to their report, the total energy from their dietary intake including alcohol and soft drinks was 2,567 ± 1,029 kcal. Of which 550 kcal were ingested from alcohol or soft drinks. Intakes of protein, fat, and carbohydrates were 68.9 ± 32.6 g, 49.2 ± 29.9 g, and 381.4 ± 171.2 g, respectively. The 2015 National health and nutrition survey reported that the total energy from the daily dietary intake was 1,942 ± 572 kcal among Japanese men aged 50–59 year [24]. That survey reported that intakes of protein, fat, and carbohydrates were 71.1 ± 21.8 g, 58.4 ± 23.7 g, and 257.9 ± 81.7 g, respectively. According to these two reports, Japanese homeless men derived higher energy from carbohydrates compared to the general population of the same age. However, the prevalence of non-communicable diseases among Japanese homeless people was similar or slightly better than that in the Japanese general population in spite of the imbalance in their dietary intake; however, there is no clear explanation for this. One of the reasons may be that most Japanese homeless people obtain income by physical labor. Suzuki [28] reported that 86.4% of homeless people derive a small income from jobs. The most common job for homeless people was the gathering of aluminum cans to sell. Although there is no data to substantiate this, it was suggested that homeless people walked generally 10 km per day to gather aluminum cans. This exercise may have had a positive impact on their health.

We also determined the relationship between participants’ socio-demographics and five non-communicable diseases (liver function abnormalities, dyslipidemia, “impaired glucose tolerance,” obesity, and hypertension). However, the findings of our study were inconclusive based on the comparisons. Our findings however showed that old homeless people (>60 years) had lower liver function abnormalities, lower obesity, and higher prevalence of hypertension than younger homeless people. Similar to a study from Poland [29], there was a statistically significant positive correlation between alcohol consumption and age (p = 0.023). However, there was no significant correlation between alcohol consumption and lower liver function abnormalities in our survey. Homeless women were more likely to have obesity and less likely to have hypertension than homeless men. Homeless people who stayed in shelter or temporary residence were less likely to have hypertension than street people. Since the prevalence of hypertension was not higher compared to the general population, and it was natural for blood pressure to increase with age, older homeless people had a surprisingly good health status in spite of their hard and dreary life.

## Limitations

The limitation of this study was that we targeted only 114 homeless people. Since the number of homeless people on the street was estimated at about 200 in Nagoya, our survey targeted 72 homeless people on the street. This number was considered adequate within the limit of possibilities available for reaching these peculiar survey participants. Furthermore, we compared our findings with other survey findings conducted under different conditions. Although we would have preferred to adjust for all participants’ conditions explored in each survey reported on the homeless people and compared in our study, the small size of our targeted population could not allow for this. Despite these limitations, this study was a very insightful report which revealed the actual health conditions of the homeless people in Japan.

## Conclusion

The percentage of homeless people in Nagoya who showed abnormalities in test values for non-communicable diseases was similar or slightly better than that reported for the 2015 national nutrition survey conducted in Japan. The results suggested that homeless people might have had more exercise from their continual daily walking, rather than the effect of a balanced and healthy nutritional habits. The findings of this study are important on the health and well-being of the homeless in Japan. The information could serve as a baseline against which further changes can be measured. Future research is necessary to monitor the changes overtime.

## Supporting information

S1 Table Relationship between participant’s personal histories, mental illness/intellectual disability, and non-communicable disease. (XLSX)
