# Correction: Relationship between non-communicable diseases and background characteristics among homeless people in Nagoya City, Japan

**DOI:** 10.1371/journal.pone.0224630

**Published:** 2019-10-24

**Authors:** Akihiro Nishio, Ryo Horita, Tadahiro Sado, Takahiro Watanabe, Ryosuke Uehara, Seiko Mizutani, Mayumi Yamamoto

There are errors in S1 Table. The fourth tab is labeled incorrectly. The correct label is: S1 Table. All other tabs were included erroneously. Please view the correct S1 Table below.

In the “Relationship between non-communicable disease and mental illness/cognitive disability” subsection of the Results, there is an error in the first sentence of the first paragraph. The correct sentence is: The results showing the relationship between non-communicable disease and mental illness/cognitive disability are shown at the end of S1 Table.

## Supporting information

S1 Table Relationship between participant’s personal histories, mental illness/intellectual disability, and non-communicable disease. (XLSX)
